# Bacterial Cellulose as Drug Delivery System for Optimizing Release of Immune Checkpoint Blocking Antibodies

**DOI:** 10.3390/pharmaceutics14071351

**Published:** 2022-06-25

**Authors:** Chih Kit Chung, Uwe Beekmann, Dana Kralisch, Katja Bierau, Alan Chan, Ferry Ossendorp, Luis J. Cruz

**Affiliations:** 1 Department of Radiology, Division Translational Nanobiomaterials and Imaging, Leiden University Medical Center, 2333 ZA Leiden, The Netherlands; c.k.chung@lumc.nl; 2 Percuros B.V., Zernikedreef 8, 2333 CL Leiden, The Netherlands; alanchan@clara.net; 3 JeNaCell GmbH, Göschwitzer Straße 22, 07745 Jena, Germany; uwe.beekmann@evonik.com (U.B.); dana.kralisch@evonik.com (D.K.); 4 Pilotality, Eerbeeklaan 42, 2573 HT Gravenhage, The Netherlands; katja@bierau.net; 5 Department of Immunology, Leiden University Medical Center, 2333 ZA Leiden, The Netherlands; f.a.ossendorp@lumc.nl

**Keywords:** bacterial cellulose, cancer immunotherapy, checkpoint blocking therapy, drug delivery system, side effects, sustained release

## Abstract

Immune checkpoint blocking therapy is a promising cancer treatment modality, though it has limitations such as systemic toxicity, which can often be traced to uncontrolled antibody spread. Controlling antibody release with delivery systems is, therefore, an attractive approach to reduce systemic antibody spread and potentially mitigate the side effects of checkpoint immunotherapy. Here, bacterial cellulose (BC) was produced and investigated as a delivery system for optimizing checkpoint-blocking antibody delivery. BC was produced in 24-well plates, and afterward, the edges were removed to obtain square-shaped BC samples with a surface of ~49 mm^2^. This customization was necessary to allow smooth in vivo implantation. Scanning electron microscopy revealed the dense cellulose network within BC. Human IgG antibody was included as the model antibody for loading and release studies. IgG antibody solution was injected into the center of BC samples. In vitro, all IgG was released within 24 to 48 h. Cell culture experiments demonstrated that BC neither exerted cytotoxic effects nor induced dendritic cell activation. Antibody binding assays demonstrated that BC does not hamper antibody function. Finally, antibody-loaded BC was implanted in mice, and serum measurements revealed that BC significantly reduced IgG and anti-CTLA-4 spread in mice. BC implantation did not induce side effects in mice. Altogether, BC is a promising and safe delivery system for optimizing the delivery and release of checkpoint-blocking antibodies.

## 1. Introduction

In the past decade, a vast amount of (pre-) clinical data have provided evidence for the therapeutic potential of immune checkpoint blockade as cancer therapy. Major breakthroughs date from 2011 and 2014, years in which Ipilimumab (anti-CTLA-4) and Nivolumab (anti-PD-1) were granted approval for clinical use, respectively [1,2]. Checkpoint blocking therapy can result in durable response rates for particular immunogenic malignancies, such as melanomas and non-small cell lung cancers. It is conceivable that checkpoint therapies might find more application also for other cancer types, and various promising therapies are already in the pipeline for further clinical evaluation. Of increasing interest are in particular combination therapies, in which, for instance, checkpoint therapies blocking distinct immune pathways are combined to harness more powerful anti-immune responses [3,4]. 

Despite this promising outlook, checkpoint blocking therapy is still confronting numerous drawbacks. Checkpoint blocking antibodies are conventionally administered systemically and in free solution. This delivery method could, however, result in the rapid spread of antibodies and consequently uncontrolled systemic activation of the immune system, conditions that usually mark the onset of immune-related adverse events (IRAEs) [5]. Furthermore, the rapid release and clearance of antibodies can lead to ineffective immune activation, as such, repeated dosing with high antibody dosing is often necessary. Not surprisingly, there is much incentive for optimizing the delivery of checkpoint-blocking antibodies, as reflected by the rapidly increasing number of studies in contemporary drug delivery and cancer immunotherapy research [6]. Multiple advances in drug delivery and cancer immunotherapy research have started to converge, the insights of which have endorsed the exploration of novel, more effective and safer approaches to harness a powerful immune response against tumors. A primary goal is to mitigate systemic side effects by releasing and confining antibodies in the vicinity of tumor-immunological organs harboring cells that can eradicate tumor cells, for instance, locally nearby the tumor [7]. 

Indeed, delivery systems such as particles, injectable hydrogels and implantable scaffolds have sparked much interest in cancer drug delivery as they bear distinct advantages that can be exploited to improve the delivery of immunotherapeutic drugs [8,9,10]. Just recently, we already demonstrated how injectable hydrogels based on poloxamer 407 could significantly reduce the systemic spread of anti-CTLA-4 antibody whilst facilitating effective tumor growth inhibition [11]. A potential consideration with injectable hydrogels, however, is their relatively rapid breakdown with the risk of premature spread of the loaded drug. This is in contrast to scaffold-like (implantable) gels, as they bear higher mechanical strength and offer more stability, which might result in even more superior control of systemic drug spread [12]. Before a scaffold is eligible as a delivery system for cancer immunotherapy, it has to satisfy a certain set of parameters: It should (1) be compatible and inert, (2) not induce inflammation or side effects and (3) be able to control drug release.

To achieve these goals, bacterial cellulose (BC) represents a potential candidate delivery system. BC can be produced by the static cultivation of *Komagataeibacter xylinus* and has already found distinct applications as wound dressing material and drug delivery system, for instance, by controlling the release of antiseptic drugs [13,14,15]. BC has a water content of >90% and possesses various beneficial properties for different medical applications. As a gel-like product, for instance, it soothes the skin and does not engraft with the skin. A recent study just reported the promising results of BC as a biomaterial implant, in which BC was tested as a cardiac implant to mimic the function as a left ventricular assist device for the treatment of congestive heart failure [16]. Recent drug delivery research has further demonstrated various other advantages of BC, including its promising effects on controlling drug release, biocompatibility, low immunogenicity and ease of production/handling. However, BC has—to date—not been studied yet as a drug delivery system for immune checkpoint blocking antibodies and, in general, have not found considerable application yet in cancer research. Based on the promises from dermal-, nano- and surgical medicine research, we reckon BC as a potential novel candidate drug delivery system that can be exploited for optimizing the delivery of immune checkpoint antibodies.

Therefore, in the current study, BC was size/shape customized, loaded with antibodies and subjected to several physico-chemical and biological characterizations. Next, in vitro antibody loading and release studies were performed. In vivo, BC loaded with IgG was implanted in mice to assess the effect on serum antibody levels. 

## 2. Materials and Methods

### 2.1. Preparation and General Characterization of BC Fleeces

BC fleeces were synthesized via the static cultivation of *Komagataeibacter xylinus* DSM 14666 (DSMZ, Braunschweig, Germany). Bacteria were cultured in 24-well plates supplied with Hestrin–Schramm medium, and the synthesized BC fleeces were harvested as reported previously [14,15,17]. Fleeces were sterilized by autoclaving (121 °C, 20 min, 2 bar) and stored at room temperature until further use. BC gross morphology and parameters, including diameter, thickness and weight were assessed. The size and shape were customized with scissors.

### 2.2. Scanning Electron Microscopy (SEM)

BC fleeces were lyophilized, and cross-sections were obtained by making diagonal incisions in the fleece. The samples were then mounted on aluminum stubs and sputter-coated with a 2 nm platinum layer. BC morphology was observed via SEM, with measurements taken at an acceleration voltage of 10 kV (NovaSEM 450). For each sample, at least three representative areas were made at a magnification of 8000×. Images were analyzed using ImageJ (National Institutes of Health, Bethesda, MD, USA).

### 2.3. In Vitro Human IgG Loading and Release Studies with Injection Method 

IgG antibodies were loaded in BC by injection, a loading method that was established by Pötzinger et al. [18]. A total of 25 µL 50 µg human IgG (Nanogam^®^; Sanquin; Leiden, The Netherlands) was injected directly into the center of a BC fleece using a 30 G U-100 insulin needle (BD Micro-Fine ^TM^, Franklin Lakes, NJ, USA). To visualize the injection depots, the injection solution was stained with Trypan blue. The loaded BCs were placed in flasks containing 5 mL 1% BSA (bovine serum albumin)/0.05% Tween-20 (Sigma-Aldrich, Steinheim, Germany) in PBS (referred to as blocking buffer). The release medium volume of 5 mL was also based on the work of Pötzinger et al. [18]. The flasks were subsequently put on an orbital shaker set at 37 °C/100 rpm and at pre-determined intervals (0, 0.5, 1, 2, 4, 6, 8, 24, 48 and 72 h), 0.5 mL of release medium was collected for IgG measurements. The sampling volume was replaced with 0.5 mL of fresh PBS to maintain sink conditions. After collecting the final timepoint, IgG concentrations were measured with an ELISA using protein A coating (Sigma-Aldrich, Steinheim, Germany) and HRP conjugated goat-anti-human IgG (Southern Biotech, Birmingham, AL, USA), as described previously by Rahimian et al. [19].

The cumulative release was calculated according to Equation (1)

*E*(%) = (*VE* Σ_1_^n^^−^^1^*C_i_* + *V*_0_*C_n_*)/*m*_0_ × 100 (1)

where *E*(%) is the cumulative release, *V_E_* is the withdrawn volume (0.5 mL), *V*_0_ is the initial volume (5 mL), *C_i_* and *C_n_* are the IgG concentrations, *_i_* and *_n_* are the sampling times, and *m*_0_ is the total amount of IgG loaded in the BC (50 µg).

### 2.4. Cytotoxicity Evaluation in MC38 Cells via 7-AAD Measurements

BC extracts were prepared to evaluate the effect of BC on cell viability. In total, 1 g of BC was incubated in 50 mL of IMDM culture medium in a flask (Lonza, BioWhittaker, #12-726F, Walkersville, MD, USA) enriched with 4% fetal calf serum (FCS; Gibco, Waltham, MA, USA), Hepes (25 mM), 2 mM L-glutamine (Gibco, Waltham, MA, USA), ß-mercaptoethanol and 100 IU/mL penicillin/streptomycin (Gibco, Waltham, MA, USA). The flasks were then put on an orbital shaker set at 100 rpm for 48 h. This method of extract preparation was established by Wiegand and colleagues for evaluating wound dressing extracts on HaCaT keratinocytes [20]. The highest extraction ratio here is 20 mg/mL, and extracts were supplied with IgG or anti-CTLA-4 antibody (Syrian hamster IgG; clone 9H10; Bioxcell, Lebanon, NH, USA) at a final concentration of 50 µg/mL. In this study, MC38 tumor cells were cultured in flat-bottom 96-well plates (5 × 10^3^/well) in the presence of increasing concentrations of BC extracts and antibodies. MC38 is a murine adenocarcinoma cancer cell line, which was established by the injection of dimethylhydrazine in C57Bl/6 mice. This tumor model has found many applications in cancer immunotherapy research, as it responds well to immune checkpoint blockade. The doubling time of the cell line is approximately half a day in vitro [21]. 

After 48 h of incubation, MC38 cells were harvested for FACS flow cytometry. Cells were detached with Trypsin/EDTA, washed with FACS buffer and stained with 7-AAD live-death cell marker (Invitrogen, Waltham, MA, USA, #A1310). After 30 min of staining, the cells were washed to remove unbound dyes and resuspended in 100 µL FACS buffer. FACS flow cytometry was performed with an LSR II cytometer (BD Biosciences, Franklin Lakes, NJ, USA), and data were analyzed using FlowJo^TM^ LLC software (Treestar Inc., Ashland, OR, USA, version 10).

### 2.5. MTS Assays for Measuring Cell Metabolism

To assess the effects of BC on cell viability, MC38 tumor cells (5 × 10^3^ cells/well) were cultured with BC extracts (as described in Section 2.4) in 96-well plates. MC38 cells were cultured in IMDM medium (Lonza, BioWhittaker, #12-726F, Walkersville, MD, USA) enriched with 4% fetal calf serum (FCS; Gibco, Waltham, MA, USA), Hepes (25 mM), 2 mM L-glutamine (Gibco, Waltham, MA, USA), ß-mercaptoethanol and 100 IU/mL penicillin/streptomycin (Gibco, Waltham, MA, USA). After 48 h of incubation, 20 µL of MTS (3-(4,5-dimethylthiazol-2-yl)-5-(3-carboxymethoxyphenyl)-2-(4-sulfophenyl)-2H-tetrazolium) reagent (CellTiter 96^®^ Aqueous One, #G3581, Promega, Madison, WI, USA) was added to the cells. To correct for the background signal, wells filled with medium only were included as well. Alive and thus metabolic active cells can convert the yellow MTS reagent to a brown end product (formazan). The resulting optical density (OD) was determined with a spectrum analyzer device (Bio-Rad, iMarkset, Hercules, CA, USA) set at 490 nm. Cell viability is expressed in percentage (%) and calculated according to Equation (2):

(*OD*490 nm *treated cells* − *OD*490 nm *background*)/(*OD*490 nm *untreated cells* − *OD*490 nm *background*) × 100 (2)

### 2.6. Dendritic Cell Maturation Studies

To assess the effects of BC on immune cells, D1DCs (a murine dendritic cell line) [22] were cultured with increasing amounts of BC extracts (up to 20 mg/mL). After 48 h, the D1DCs were detached using PBS/EDTA, washed with FACS buffer (PBS with 0.5% BSA and 0.02% sodium azide) and stained at 4 °C for 30 min with the following antibodies: anti-CD40-APC (Biolegend, San Diego, CA, USA, clone 3/23, #12-4612), anti-CD86-FITC (eBioscience, San Diego, CA, USA) clone GL1, #11-0862-85,) and anti-MHC II-PE (eBioscience, San Diego, CA, USA) clone M5/114.15.2, #12-5321-82). 7-AAD (Invitrogen, Waltham, MA, USA, #A1310) was included to stain the dead cells. Thereafter, the cells were washed with FACS buffer to remove unbound antibodies and resuspended in 100 μL FACS buffer. FACS analysis was performed on an LSR-II cytometer (BD Biosciences, Franklin Lakes, NJ, USA). The data were analyzed with FlowJo V10. 

### 2.7. Antibody Binding Assessment In Vitro

Utilizing an assay that is based on a two-step antibody staining, we assessed whether the anti-PD-L1 checkpoint blocking antibody retained its binding capacity after being incorporated in BC. For this assay, anti-PD-L1 antibody (rat IgG, clone 10F.9G2, Bioxcell, Lebanon, NH, USA) was loaded in BC. After 7 days, the released antibodies were collected. Anti-PD-L1 was added to B16F10 cells (murine melanoma cell line) which were pre-stimulated twenty-four hours with 10 IU/mL IFN-γ (Thermofisher, Waltham, MA, USA). Next, a secondary antibody—Alexa 488 donkey-anti-rat IgG (Thermofisher, Waltham, MA, USA)—was added as staining. The fluorescence intensity of the Alexa 488 is indicative of the amount of bound anti-PD-L1 and was measured on an LSR-II cytometer (BD Biosciences, Franklin Lakes, NJ, USA). Data were analyzed with FlowJo V10.

### 2.8. BC Implantation in Mice and In Vivo Release Studies

BC fleeces were loaded with human IgG antibody (50 µg) or anti-CTLA-4 (Syrian hamster IgG, clone 9H10; Bioxcell, Lebanon, NH, USA; 100 µg) according to Section 2.3. An incision of approximately 10 mm was made on the right flank of C57BL/6j mice and the BC fleeces were carefully put under the skin of the mice. The wounds were closed with a combination of surgical reflex clips (7 mm) and resorbable 5-0 vicryl suture (Ethicon, Raritan, NJ, USA, #V303H). Blood was drawn and subjected to IgG measurements according to Section 2.3, 1, 2, 3, 7 and 14 days after implantation. C57BL/6j mice were purchased from Janvier Labs (Le Genest-Saint-Isle, France) and housed under pathogen-free conditions in the animal facility of the Leiden University Medical Center (LUMC). The Animal Welfare Body (AWB) and Animal Experimental Committee (DEC) from the LUMC approved all experiments, which were subjected to the Dutch laws of animal experimentation and conducted in accordance with the regulations as stated in animal project license number AVD11600202010185. 

### 2.9. Statistical Analysis

Data and graphs were prepared using GraphPad Prism 8.0.1 (244) (San Diego, CA, USA) and Adobe Illustrator 2021 (San Jose, CA, USA). Where applicable, the types of statistical tests performed are mentioned in the figure legends. Data are shown as mean ± standard deviation (SD). *p* values < 0.05 are regarded as statistically significant. N.S. denotes not significant. 

## 3. Results

### 3.1. BC Gross Morphology and Size Customization 

**Figure 1:**
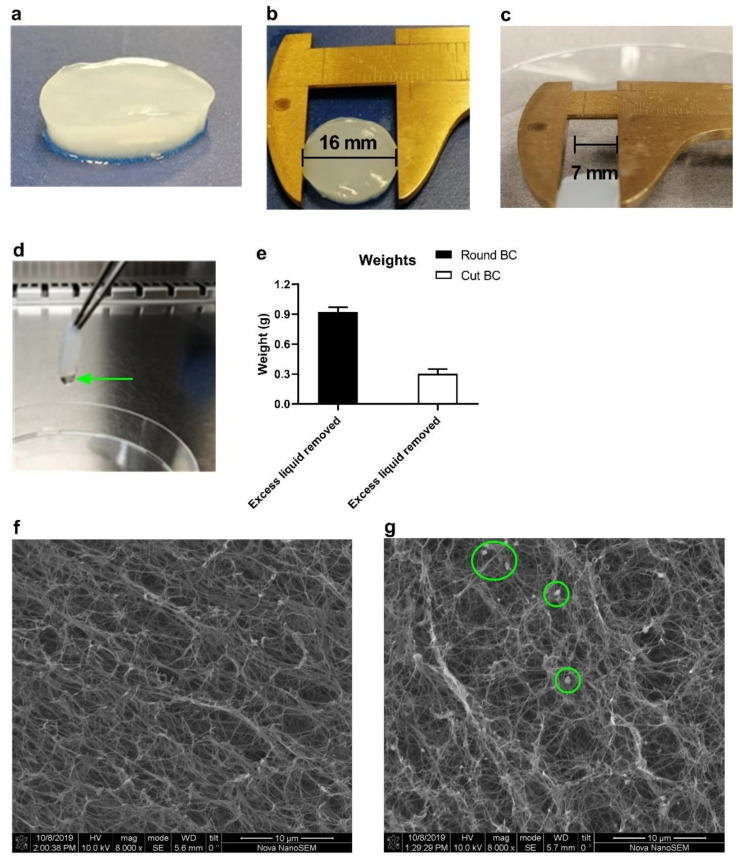
Characteristics and morphology of bacterial-derived cellulose (BC). (**a**,**b**) The photographs show the appearance of a freshly produced (native) BC fleece (referred to as round BC), which has the shape and size of the wells of a 24-wells culture dish. Image (**c**) shows a fleece of which the edges were cut (cut BC). Excess liquid droplets were removed by holding the BC fleece with forceps (**d**), and afterward, the fleeces were weighted (**e**). Figures (**f**,**g**) represent representative SEM images for native and IgG-loaded BC, respectively. Data in (**e**) are shown as mean ± SD for triplicate measurements.

BC was produced by the static cultivation of Komagataeibacter xylinus in 24-well plates. The harvested gel-like product consists of a 3D cellulose matrix of cellulose fibers with a spherical shape, which firmly binds a significant amount of water (Figure 1a). This original, freshly produced BC (referred to as round BC; Figure 1b) has a diameter of 16 mm and thickness of 3 mm. Its size and shape are easily customized using scissors. The objective of this study was to obtain BC samples with a size and shape that would allow smooth implantation in mice. In this regard, we considered that a BC sample with a surface of 49 mm^2^ (7 × 7 mm) and a thickness of 3 mm meets these requirements (here onwards referred to as cut BC; Figure 1c). 

Subsequently, the BC gels were weighted. Excess fluid was removed by holding the fleece with forceps; this allowed excessive amounts of water drops to build and fall off. This procedure was repeated until no droplets were forming (Figure 1d). Round BC weighted on average 0.9 g, whilst cut BC weighted 0.3 g after excessive fluid removal (Figure 1e). The SEM image of Figure 1f clearly shows the densely interwoven cellulose fiber network of native BC. Loading with antibodies did not affect this structure, although some salt depots (marked with circles in Figure 1g; derived from the in PBS prepared antibody solution) were visible.

### 3.2. IgG Loading and Release Kinetics In Vitro

**Figure 2:**
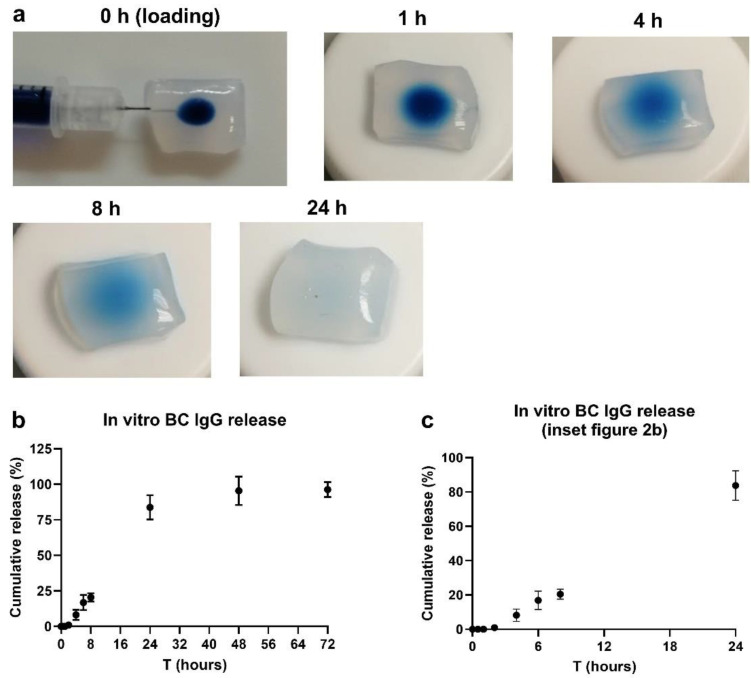
Human IgG loading and in vitro release were performed with the ‘injection method’. (**a**) A total of 50 µg IgG in a volume of 25 µL PBS was injected into the center of a BC fleece. To visualize the injection depot, the IgG solution was supplied with Trypan blue. The loaded fleeces were then put in blocking buffer (1% BSA/0.05% Tween-20 in PBS) and IgG release was followed. At several time-points, the fleeces were taken out, and the Trypan blue injection spots were photographed. Graph (**b**) shows the cumulative IgG release in %, with the inset of the first 24 h shown in (**c**). Release data are shown as cumulative release, which are displayed as mean ± SD for a triplicate measurement.

Several loading techniques can be considered for BC. Initially, methods such as ‘soaking’ and ‘injection’ (as described by Pötzinger and colleagues [18]) were considered candidates. However, the clear disadvantage of the ‘soaking method’ is the low loading efficiency (~10%), meaning that at least 1000 µg of antibody would be required to load 100 µg (Supplement Figure S1). Regarding the very high costs of many checkpoint blocking antibodies, it was thus necessary to consider an alternative technique with higher loading efficiency. Preliminary experiments furthermore showed that release was incomplete (~60%), whilst the variation in the data was large. Several technical issues (antibodies still being entrapped in the BC, etc.) might underly the poor release kinetics with the soaking method. Additional studies are thus needed to solve these technical issues, which falls outside the scope of the current research. Taken together, the injection method was considered the most practical and efficient technique for antibody loading. The release studies were initially all optimized with a human IgG antibody (Nanogam^®^), which is considered an economical and representative model antibody (comparable size as most immune checkpoint antibodies) for optimizing release kinetics studies [11,19]. IgG injection solutions were prepared and stained with Trypan blue dye, which allows the visualization of the injection depot. The loaded BC fleeces were then placed in release medium. At several time-points, BC fleeces were taken out from the release medium, and the injection depots were photographed. As becomes evident from Figure 2a, the color intensity gradually diminished over time, with almost all the signals being gone after 24 h. The rate of color change corroborates the IgG release kinetic data, as shown in Figure 2b. Figure 2c is an inset of Figure 2b, showing the first 24 h of release. From the release kinetic graph, it is evident that ~80% of the IgG is released around 24 h, with the remainder being released between 24 and 72 h. 

For the analysis of the release kinetics in Figure 2b, several mathematical models have been considered. Eventually, the zero-order kinetic model resulted in the best fit, which can be described with the equitation Y = 3.58x − 4.070 and an R^2^ of 0.976. This means that the amount of drug released increases linearly over time (with a release constant of 3.58 per hour) until reaching the plateau, which was at 24 h in this experiment. 

### 3.3. BC Extracts Do Not Induce Cytotoxic Effects In Vitro 

**Figure 3:**
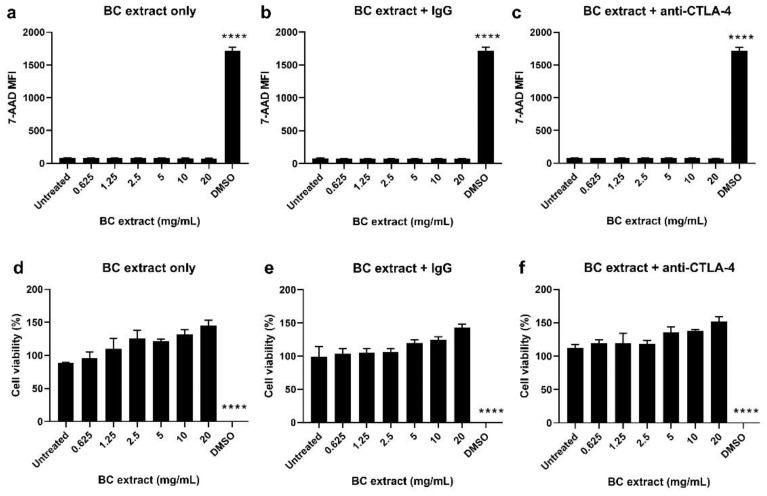
Cytotoxicity evaluation of BC extracts in MC38 tumor cells with 7-AAD staining and MTS. BC extracts were prepared by dissolving 1 g of BC in 50 mL culture medium, which yielded an extraction ratio of 20 mg/mL. Empty extracts or extracts supplied with human IgG or anti-CTLA-4 (both starting at a concentration of 50 µg/mL) were tested on MC38 cells. DMSO was used as positive cell killing control. The primary measure was cell viability, which was assessed by 7-AAD staining (live-dead cells exclusion marker; figures **a**–**c**). Higher 7-AAD MFI signal is a hallmark of dying cells with leaky cell membranes. Cytotoxicity was also assessed using a cell metabolism assay (MTS), in which higher cell metabolism is indicative of higher cell viability (**d**–**f**). Cell metabolism was measured 48 h after incubation. All data are shown as mean ± SD for triplicates. Only the DMSO treatment significantly decreased cell viability, which was assessed with a Student’s *t* test, with **** denoting *p* < 0.0001.

Due to the shape and solid structure of the BC fleeces, it is practically difficult to directly assess their effects on cell viability in a cell culture experiment. A possible approach is to incubate BC in culture medium for 48 h and add this ‘BC extract’ to cells. This setup is based on the method described by Wiegand et al. [20], in which the effect of BC extracts was evaluated on HaCaT keratinocytes. In the current study, the effect of BC extracts (supplied with antibodies) on cell viability was evaluated on MC38 colon carcinoma cells. Figure 3a–c shows the results of 7-AAD staining. As can be seen, BC extracts or BC extracts supplied with either IgG or anti-CTLA-4 antibody up to a concentration of 20 mg/mL did not induce any increase in the 7-AAD signal, unlike the DMSO assay control (*p* < 0.0001). This result indicates that BC extracts did not induce cytotoxic effects. This result is supported by cell metabolism measurements (MTS) assays. The graphs in Figure 3d–f show that the BC extracts or BC extracts supplied with antibodies did not significantly reduce MC38 cell metabolic activity, as opposed to the DMSO control (*p* < 0.0001). In conclusion, using both 7-AAD staining and cell metabolism assays, we demonstrated that BC extracts do not compromise cell viability in vitro.

### 3.4. BC Extracts Do Not Induce Expression of DC Activation Markers

**Figure 4:**
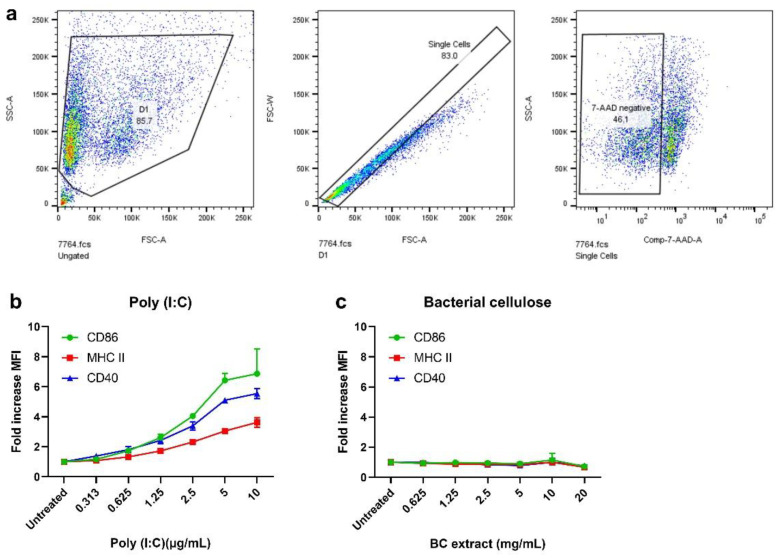
BC extracts do not induce DC maturation. BC extracts (max concentration 20 mg/mL) were co-cultured with D1DCs. After 48 h, D1DCs were harvested to assess the expression of CD40, CD86 and MHC-II activation markers with FACS. Debris, doublet cells and dead cells (7-AAD positive) were gated out (**a**). Next, the fold increases in MFI (the MFI of treated cells relative to untreated controls) were calculated for poly (I:C) (**b**) and BC extracts (**c**). Data are shown as mean ± SD for triplicate measurements.

Before a delivery system can be considered for cancer immunotherapy, it is crucial to assure that it is inert, free of (endo) toxins and does not trigger inflammation. To assess these criteria, D1DCs were co-cultured with BC extracts for 48 h. Afterward, the cells were harvested to assess the expression of CD40, CD86 and MHC-II. The expression of these markers usually increases in the presence of immunostimulatory or potentially harmful (danger) signals. After gating out doublet cells and 7-AAD+ cells (Figure 4a), expression of CD40, CD86 and MHC-II on BC-treated D1DCs was measured and compared with D1DCs treated with poly (I:C) (TLR3-Ligand, positive control). As can be seen, BC did not lead to increased expression of any of these markers, as compared to poly (I:C) (Figure 4b,c). In addition to being biocompatible, this assay thus provides a clear indication that BC is inert, likely free of contaminants and not immunogenic. 

### 3.5. Antibody Binding Capacity Is Not Affected by BC

**Figure 5:**
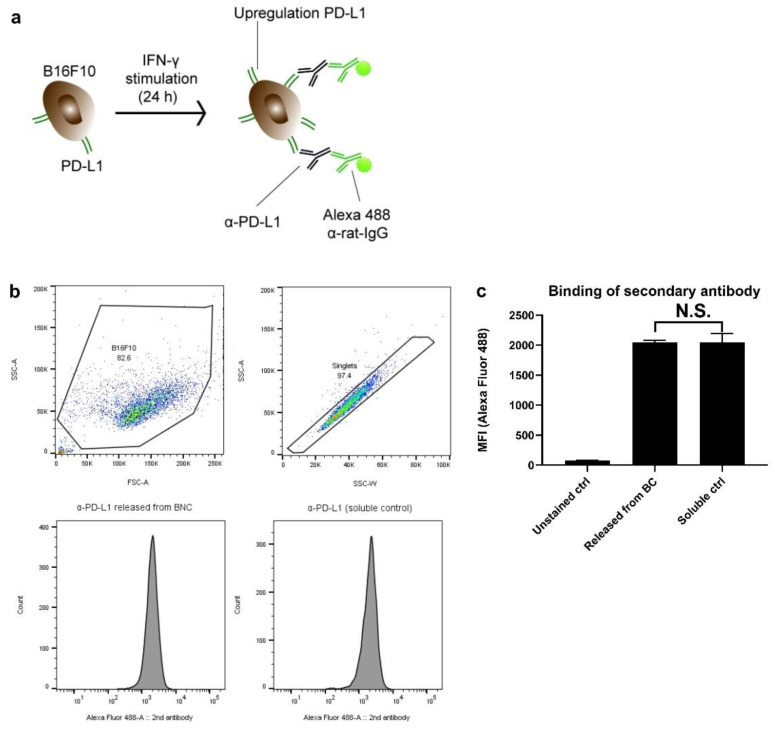
BC does not affect anti-PD-L1 binding capacity to B16F10 cells. In total, 50 µg of anti-PD-L1 in 25 µL PBS was injected in a BC fleece. After 7 d, the released antibodies from the BC were harvested to assess the binding capacity. B16F10 cells were incubated 24 h with 10 IU/mL IFN-γ to upregulate PD-L1 expression. Thereafter, the cells were harvested, washed and incubated with the anti-PD-L1 that was released from the BC samples. In the subsequent step, cells were stained with fluorescent labeled anti-rat IgG (Alexa 488 donkey-anti-rat IgG) (**a**). Alexa 488 fluorescence was a measure for PD-L1 binding. This was compared with cells to which stock (fresh) of anti-PD-L1 antibody was added (‘soluble control’). In (**b**), the gating strategy and representative histograms with the Alexa 488 emission peaks are displayed, with the quantification of the mean fluorescent index (MFI) depicted in (**c**). In (**c**), data are shown as mean ± SD for a triplicate measurement, with differences being assessed with Student’s *t* test and N.S. denoting ‘not significant’.

Figure 5a depicts in chronological order the staining process. The fluorescence intensity of the Alexa 488 is indicative of the amount of bound anti-PD-L1. Figure 5b shows the gating strategy (debris and doublets gated out), whilst the graphs below are representative histograms displaying Alexa 488 fluorescence intensity. As assay control, untreated antibody (fresh from the stock; referred to as ‘soluble control’) was included. As can be seen in Figure 5c, the fluorescence intensity for cells that were co-incubated with the ‘BC released antibodies’ remained as high as cells stained with the ‘soluble control’ (n.s. denoting no statistical differences). These data indicate that BC does not affect anti-PD-L1 antibody binding efficacy. 

### 3.6. BC Reduces Peak Serum Level of IgG in Mice

**Figure 6:**
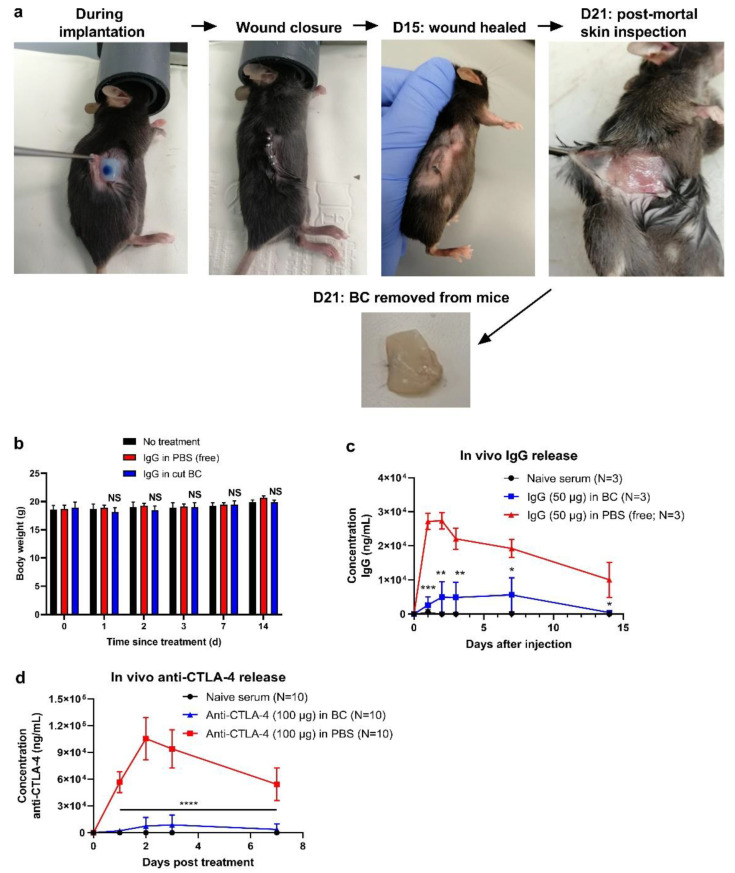
BC reduces serum antibody levels in vivo. Photographs in (**a**) depict in chronological order the procedures of the BC implantation, starting with loading, placing the BC sample underneath the skin, wound closure, visual inspection of the skin and removing the BC implant at D21 (after the mice were killed). Body weight measurements are depicted in (**b**), for the BC-treated mice, the body weight differences at various time-points were compared with the initial body weight at D0. Body weight differences were assessed with a paired Student’s *t*-test, with NS denoting no statistical differences compared to the body weights at D0. In (**c**) and (**d**), the serum IgG and anti-CTLA-4 levels are shown for several time-points after treatment, respectively. Statistical differences between the BC and PBS group were assessed with an unpaired Student’s *t*-test and are denoted as * *p* < 0.05, ** *p* < 0.01, *** *p* < 0.001 and **** *p* < 0.0001.

Having satisfied the most crucial parameters in vitro, we sought to evaluate the antibody-loaded BC in vivo. To date, this is the first study in which antibody-loaded BC is tested as an implantable delivery system in mice. Figure 6a is a chronological representation of the study, starting with the implantation, followed by wound closure (using reflex clips and sutures) and visual monitoring of the mice. Surgical clips could be removed after 10 days, with the wounds being completely healed on day 15. At the end of the study (D21) mice were sacrificed. The BC was removed, and the skin of the mice was visually inspected. As can be seen, the blue color of the injection depot is gone, suggesting that all IgG has been released in vivo. Compared to pre-implantation, the BC did shrink a bit in size as the water content has likely been absorbed after implantation. It is furthermore apparent that BC treatment did not cause irritation to the skin. Another good indicator of biocompatibility is body weight. As can be seen in Figure 6b, BC-treated mice maintained a constant body weight over time (compared to the initial weight at D0), indicating that the BC treatment did not compromise the animals’ health. This observation is thus in line with the results of the in vitro biocompatibility measurements. 

At D1, 2, 3, 7 and 14 after implantation, blood was drawn to determine serum IgG concentration. IgG levels were compared to mice that received the IgG in free solution (PBS; Figure 6c). As can be clearly seen, BC hydrogels significantly reduced IgG levels (between days 1–3), as compared to mice that received IgG in PBS. Between day 7 and 14, IgG levels started to decline in both groups, although this observation is most likely ascribed to the average half-life of IgG in vivo (6 to 8 days) [23]. In another experiment, anti-CTLA-4 release was also assessed. BC samples were loaded with 100 µg anti-CLTA-4 and implanted in mice according to the IgG study. At 1, 2, 3 and 7 days after treatment, blood was sampled and subjected to anti-CTLA-4 measurements. In line with the IgG serum measurements, the anti-CTLA-4 serum analysis revealed that BC significantly reduced the systemic spread of antibodies as compared with delivery in PBS (Figure 6d). The release pattern is comparable with that of the IgG serum analysis (Figure 6c). Altogether, these data suggest that BC gels strongly reduce the systemic spread of antibodies in vivo, which indicates the reduction in systemic side effects. 

## 4. Discussion

Checkpoint blocking therapy can be effective for the treatment of immunogenic tumors, such as melanomas that bear neo-antigens as a result of many (point) mutations. However, systemic side effects as well as rapid antibody spread and clearance are pitfalls that warrant the exploration of delivery systems that can optimize the delivery of checkpoint blocking antibodies. In recent years, promising results from drug delivery research have provided rationale to optimize cancer drug delivery with the aid of, for instance, hydrogel and scaffold-like delivery systems. 

The current study has, for the first time, exploited BC as a delivery system for optimizing the delivery of checkpoint blocking antibodies. Herein, BC was loaded with antibodies and release profiles were studied in vitro and in vivo. Furthermore, various physico-chemical parameters and biological effects were studied. We deem the results of this study as valuable starting points for the design of safer immune checkpoint blocking therapies. 

BC was produced in 24-wells plates according to existing protocols. Since the eventual goal was to assess BC as an implantable scaffold in vivo, it was necessary to establish a protocol for the implantation procedure. We customized the size and shape in such a way that (1) BC can be implanted smoothly and (2) BC can be easily prepared in a standardized manner. It was envisaged that square-shaped BC with a maximum area of ~49 mm^2^ and a thickness of 3 mm is fulfilling these criteria. Square BC was obtained by cutting out the round edges of freshly produced BC, which was an easily reproducible technique. SEM images clearly demonstrated the fiber-like network within BC, the structure of which was not altered by the injection of antibodies. The morphology is comparable with what was observed in previous studies. The antibody-loaded BC did show some salt agglomerations on the fibers. This observation was, however, also reported before by Müller et al. [24], which is likely ascribed to the fact that the IgG injection solution was prepared in saline buffer. The dense network is possibly responsible for retaining the fluid within the BC. The exact distance between the fibers (pore size) is hard to determine accurately since the cellulose network density varies, but it is generally estimated to be ~5 µm for native (unmodified) BC [25]. 

Several established techniques were considered for antibody loading, including loading by soaking, injection or immersion of lyophilized BC. Regarding the high costs of checkpoint blocking antibodies, we opted for a method that ensures high loading efficiency and that is technically easy to reproduce. This was attainable with the injection method, as the antibodies can be loaded quickly and easily, without any loss of antibodies. In vitro release kinetic studies showed that BC released ~80% of the loaded IgG within 24 h, with the remaining 20% being released within 48 h. With 100% of the antibodies being released, it can indeed be concluded that no antibodies were lost using the injection method. It can be conceived that the IgG release followed a constant release (zero-order) kinetic with a fairly linear increase in drug release. This is in contrast with the study of Pötzinger et al., in which the authors loaded BC with pSV-β-Gal and pGL3 plasmids. The authors found the best fit with the Rittger–Peppas model and described that both non-Fickian and Fickian diffusion play a role [18]. Regarding the size and weight of antibodies (150 kDa), there is also a possibility that the release here was more facilitated by the constant rate of dissolution of the injection depot in the release medium. Such effect was indeed observed in the work of Nie et al. [26], in which the authors studied paclitaxel release from poloxamer hydrogel depots. The linear increase in paclitaxel release (zero-order) was directly correlated with the linear rate of hydrogel dissolution. Additional studies would be interesting to compare the effect of size on release kinetics utilizing the injection method. In general, the zero-order release kinetic could be considered as beneficial as this could help to keep drug plasma levels constant, minimizing toxicity and circulation time of the antibody [27,28]. 

To evaluate the biological effects, BC extract was prepared and co-incubated with different cell types. This is an indirect approach to assess how cells respond to BC samples. It is technically difficult to co-incubate BC implants with cells as the cells might be soaked into the BC sample. BC extracts were prepared according to a protocol established by Wiegand et al. [20]. The extracts were supplied with IgG and anti-CTLA-4 antibodies and were added to different cell types. With both 7-AAD (death marker) and MTS (cell metabolism), it was shown that the BC extracts, or BC extracts mixed with IgG/anti-CTLA-4, exerted no negative effects on cell viability. These observations are in line with another study by Wiegand et al. [15], in which the authors showed that BC exerts no cytotoxic effects on human keratinocytes. 

In addition to ensuring that BC is biocompatible, it is important to assure that the material is inert. It should neither induce inflammation nor contain (endo) toxins. Incubating DCs with a test compound and assessing for inflammatory marker expression could provide indications of whether these criteria have been met. Here, D1DCs were incubated with BC extracts. After 48 h, expression of CD40, CD86 and MHC-II—markers that are indicative of DC maturation [29]—was measured. None of the BC extract concentrations induced the expression of any DC marker. This result indicates that BC has neither inflammatory nor immunogenic properties. Furthermore, it indicates that the material likely free of compounds or endotoxins might trigger immune cell activation. Altogether, the results of the cell viability studies and DC activation support the notion that BC is non-toxic and non-immunogenic. 

Studies reported that BC does not hamper drug function, as has been shown with, for instance, antiseptic drugs [14,30]. In the current study, we demonstrated that anti-PD-L1 antibodies, after being released from BC, maintained the same binding efficacy as fresh antibodies derived from the stock. This means that BC does not affect the antibody binding efficacy, the result of which is in line with previous studies in which researchers demonstrated that antiseptic drugs still maintained antimicrobial activity after being released from BC [31]. 

Having satisfied these in vitro parameters, we established an implantation protocol in mice to evaluate the BC as a delivery system in vivo. Blood was collected to assess antibody levels. Only limited literature is available regarding the in vivo BC drug release kinetics. The implantation lasted approximately 10 min and was minimally invasive with a small skin incision of ~10 mm. During the first 2 h after implantation, it was apparent that the mice recovered quickly. Wounds already healed fully in 10 days and post-mortal analysis showed that the skin neither showed redness nor inflammatory lumps. These clinical signs were used as criteria because mice treated with, for instance, oil-based emulsions (including Montanide or Freund’s Adjuvant) typically manifest these inflammatory signs [32,33]. Overall, the positive observations in our study corroborate those of earlier studies, which mainly reported that BC is a biocompatible biomaterial that soothes the skin, promotes wound healing, can stimulate tissue regeneration and does not induce inflammation [34,35,36,37,38]. Blood serum analysis after implantation showed that BC significantly reduced IgG as well as anti-CTLA-4 peak levels, as compared to mice that received IgG in free solution (more than 5-fold), which is a considerable factor of decrease in cancer drug delivery studies. This result is comparable with what was found in a comparable study, in which a thiol-modified hyaluronic acid (Glycosil) based scaffold was developed for the sustained release of anti-CTLA-4 and anti-PD-1 antibodies. Using imaging studies, the authors found that hydrogel scaffolds retained the signal of fluorescent-labeled antibodies for a prolonged period. Twenty-four hours after implantation, the fluorescence signal in the hydrogel group was three times higher compared to the group receiving freely administered antibody, which implies that a three-fold higher amount of antibody was confined at the implantation site [39]. There is a strong correlation between systemic antibody levels and the induction of, for instance, IRAEs, since the receptors of these target antibodies are expressed in various tissues in the body [40]. Regarding this correlation, it can be concluded that BC potentially mitigates the side effects of checkpoint-blocking immunotherapy. 

Altogether, the in vivo antibody release kinetics are promising, and the implantation did not compromise animal welfare. In a pilot experiment, we sought to evaluate whether BC loaded with anti-CTLA-4 antibodies can efficiently inhibit tumor growth in the MC38 colon cancer model, which is sensitive to CTLA-4 therapy. Mice were treated with BC loaded with 100 µg anti-CTLA-4. This anti-CTLA-4 dosing range (50 to 100 µg) is based on earlier studies with the same tumor model. Preliminary data, however, showed that with the current setup, 50 to 100 µg BC CTLA-4 blockade did not result in tumor growth inhibition (data not shown). Additional studies should focus on finding the optimal conditions to exploit BC for tumor treatment, as it is unknown whether other factors (apart from the antibody concentration or release) affect treatment outcome. The relationship between anti-CTLA-4 antibody serum concentration and treatment outcome could be ambiguous. In the study of Rahimian, for instance, mice were treated with a single shot of 50 µg anti-CTLA-4 and anti-CD40 in microparticles. Serum analysis revealed an almost 10-fold reduction in antibody concentration, which was comparable with what we observed. The low systemic antibody concentration did, however, result in effective MC38 tumor growth inhibition [19].

From the surgical point of view, it is possible that the wound healing promoting properties of BC—as well as the wound healing factors secreted by the wound—exert adverse effects on immune cell activation, especially cells that play a central role within the TH1-immune axis. Although accelerated wound healing promotes quick recovery of the surgical wound, it might also suppress anti-tumor immunity [41,42,43,44]. In the study of Park et al. [39], the authors also designed various anti-cancer therapies based on implantable scaffolds. Anti-CTLA-4 and anti-PD-1 loaded scaffolds were implanted directly at the surgical site after the removal of orthotopic 4T1 breast cancer tumors. Remarkably, even with a combination of anti-PD-1 and anti-CTLA-4 at a dose as high as 300 µg each, the scaffold-based treatment failed to effectively inhibit tumor growth. On the other hand, treatment with certain adjuvants, such as TLR7/8 or STING agonist, did result in effective tumor growth inhibition. It remains to be explored whether BC therapies with certain other immunotherapeutic combinations could exert more meaningful effects on repolarizing the post-surgical environment into one that has immunostimulatory and anti-tumor properties, with the goal of conferring meaningful effects on tumor control. Specific drug combinations—such as checkpoint therapy with cancer vaccines, or immune adjuvants with chemotherapeutics—might be further considered to attain this goal [45,46,47,48,49].

The antibody binding assays demonstrated that the antibodies incorporated in the BC fleeces maintained their binding capacity, as compared to antibodies from a stock solution (Figure 5). To rule out other technical issues, we performed a post-mortal analysis whereby the BC was removed 14 d after implantation. The contents were extracted to analyze IgG content; however, all samples remained negative, which ruled out the possibility that the antibodies were still trapped inside the BC (data not shown). 

Thus, data from this study support the notion that BC can be a promising delivery system for checkpoint-blocking antibodies. BC releases checkpoint-blocking antibodies in a sustained manner in vitro and in vivo. Furthermore, BC did not induce cytotoxic effects in cell culture, nor did it compromise animal welfare. Tailor-made BC may offer promising outlooks in the field of cancer research. Future research might focus on how BC treatment should be modulated in such a way that it could further improve drug release kinetics in vivo whilst exerting stronger therapeutic effects on the tumor. A possible approach is to vary the thickness to volume ratio of the BC insert by using chemical modification techniques or an adapted pore size, which might facilitate even better release in vivo [50,51]. Moreover, as BC can significantly lower the systemic spread of drugs and thus possibly lower the risk of side effects, it would be interesting also to test different drug combinations as there might be room to explore combination therapies that might be difficult to test in the current clinical settings.

## Data Availability

Data are available upon request.

## Supplementary Materials

The following supporting information can be downloaded at: https://www.mdpi.com/article/10.3390/pharmaceutics14071351/s1, Figure S1: Human IgG loading with the ‘soaking’ method.

## Abbreviations

BC (bacterial cellulose); BSA (bovine serum antigen); CTLA-4 (cytotoxic T lymphocyte antigen); DC (dendritic cell); EDTA (ethylenediaminetetraacetic acid); ELISA (enzyme-linked immunosorbent assay); FACS (fluorescence-activated cell sorting); FCS (fetal calf serum); HRP (horseradish peroxidase); IMDM (Iscove’s Modified Dulbecco Media); IRAE (immune related adverse event), PD-L1 (programmed death-ligand 1), SEM (scanning electron microscopy); TLR (Toll-like receptor).
