# Supplementary figures and images for: Bacterial Cellulose as Drug Delivery System for Optimizing Release of Immune Checkpoint Blocking Antibodies
Source: Pharmaceutics. 2022 Jun 25;14(7):1351. doi: 10.3390/pharmaceutics14071351 (PMC9316226; doi:10.3390/pharmaceutics14071351)

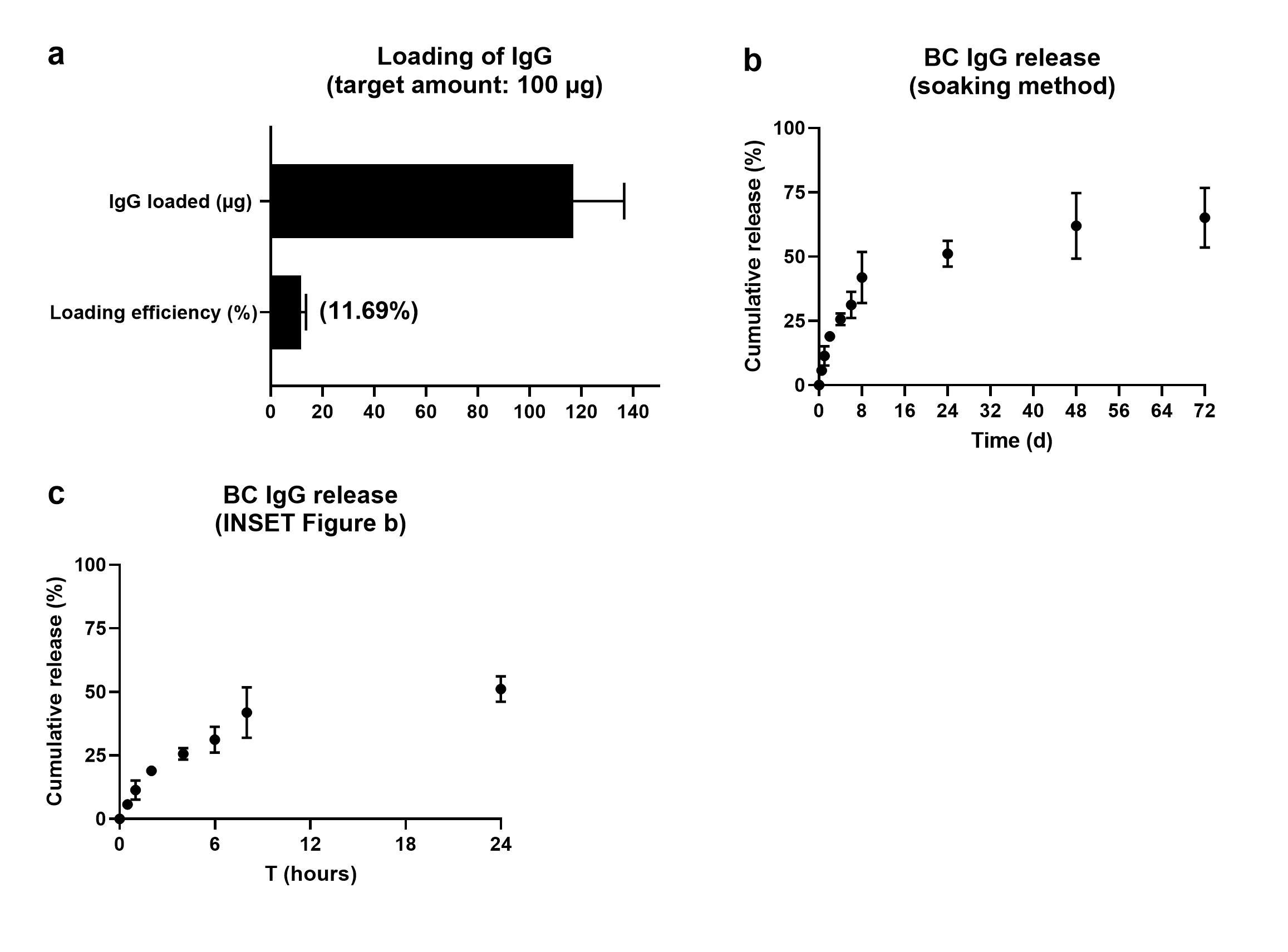

Supplement: Supplementary file 1 [file pharmaceutics-14-01351-s001.zip › Figure S1 - revised.jpg]
